# Achieve High Dielectric and Energy-Storage Density Properties by Employing Cyanoethyl Cellulose as Fillers in PVDF-Based Polymer Composites

**DOI:** 10.3390/ma16124201

**Published:** 2023-06-06

**Authors:** Deqi Wu, Mingxuan Luo, Rui Yang, Xin Hu, Chunhua Lu

**Affiliations:** 1 College of Materials Science and Engineering, Nanjing Tech University, Nanjing 211800, China; 202061103047@njtech.edu.cn (D.W.); 202061103037@njtech.edu.cn (M.L.); 202261103077@njtech.edu.cn (R.Y.); 2 State Key Laboratory of Materials-Oriented Chemical Engineering, Nanjing Tech University, Nanjing 211800, China; 3 Jiangsu National Synergetic Innovation Center for Advanced Materials (SICAM), Nanjing 211800, China

**Keywords:** polyvinylidene fluoride, graft modification, cyanoethyl cellulose, composites, polymer dielectrics

## Abstract

Fluoropolymer/inorganic nanofiller composites are considered to be ideal polymer dielectrics for energy storage applications because of their high dielectric constant and high breakdown strength. However, these advantages are a trade-off with the unavoidable aggregation of the inorganic nanofillers, which result in a reduced discharge of the energy storage density. To address this problem, we developed polyvinylidene fluoride (PVDF) graft copolymer/cellulose-derivative composites to achieve high-dielectric and energy-storage density properties. An enhanced dielectric constant and improved energy density were achieved with this structure. The optimal composites exhibited a high discharge energy density of 8.40 J/cm^3^ at 300 MV/m. This work provides new insight into the development of all-organic composites with bio-based nanofillers.

## 1. Introduction

To meet the increasing demand for compact and efficient electric systems, dielectrics with high dielectric and energy-storage density properties are required for dielectric capacitors. Polymers that are lightweight, flexible, easy to process, low cost and with a high breakdown strength (*E*_b_) are preferred in the application of dielectrics. Polyvinylidene fluoride (PVDF)-based fluoropolymers with extra advances in high permittivity (*K*) have attracted much attention as promising polymer dielectrics [1,2,3,4]. However, PVDF-based ferroelectric polymers have large remnant polarization (*P*_r_) and a low saturated electric field, which lead to a low discharge energy density (*U*_e_), greatly limiting its application in dielectric capacitors [5,6,7]. In order to obtain a higher *U*_e_, a variety of strategies have been developed to modify the microstructures of PVDF-based polymers and reduce their *P*_r_. Among these strategies, the modification of PVDF-based polymers by incorporating functional segments into the side chain via improved atom transfer radical polymerization (ATRP) has proved to be a feasible and facile method [8,9,10,11]. Conduction loss and *P*_r_ are suppressed by inducing the side chain in PVDF-based polymers [12], which can theoretically increase the energy storage density.

However, the maximum polarization (*P*_max_) also declines with a reduction in the *P*_r_, which is responsible for a decrease in the energy storage density. *P*_max_ is closely related to permittivity as *P*_max_ = *ε*_0_*ε*_r_*E*_b_, where *ε*_0_ and *ε*_r_ are the dielectric constant of the vacuum and dielectric, and *E*_b_ is the applied electric field [13,14,15]. Therefore, in order to achieve high-performance dielectrics, the development of composites to combine the advantages of high *K* fillers and high *E*_b_ PVDF-based polymers has been widely explored to enhance the energy storage density. Ceramic nanofillers (*K* > 10^2^), including but not limited to BaTiO_3_ [16,17], Ba_0.6_Sr_0.4_TiO_3_ [18,19], Na_0.5_Bi_0.5_TiO_3_ [20], TiO_2_ [4,21], are usually utilized to elevate the *K* of composites. Nevertheless, the great difference in the surface energy between inorganic nanofillers and an organic matrix often leads to the aggregation of the fillers, which is responsible for the deteriorated energy storage density [22]. As the most abundant bio-based polymer on Earth, cellulose has gained increasing interest in research because of its features of sustainability, thermal stability, low cost, etc. However, cellulose is nonsoluble in convent organic solvent due to the strong inter- and intramolecular hydrogen boding. Cyanoethyl cellulose (CEC) is one of the most widely investigated cellulose ethers that is prepared by cyanylation of cellulose. It exhibits a high *K* and good solubility in organic solvent [23,24]. CEC with a high degree of substitution also has a high dielectric constant and relatively low dielectric loss. Employment of CEC as fillers for dielectric composites would offer the composites a high constant and good dispersivity, which is essential to the dielectric and energy storage properties [25,26].

In this work, we developed polyglycidyl methacrylate (PGMA)-functionalized PVDF-based copolymer/CEC composites to improve the compatibility and dielectric constant simultaneously to obtain all-organic composite dielectrics. Enhanced energy densities were also accessed in this structure, which may be ascribed to the hydroxyl groups on CEC, increasing the interaction with the polymer matrix. This work contributes to the application of bio-based polymers serving as fillers in composites to yield high-performance dielectrics.

## 2. Materials and Methods

### 2.1. Materials

P(VDF-*co*-HFP) was purchased from Arkema Company (Shanghai, China, Arkema Kynar 2801, with the [VDF]:[HFP] = 90:10). Glycidyl methacrylate (GMA, 97%) was obtained from Aladdin Reagents (Shanghai, China). 10-Phenylphenothiazine was purchased from Shanghai Yien Chemical Technology Co., Ltd. (Shanghai, China). Cyanoethyl cellulose was purchased from Beijing North Century Cellulose Technology Development Co., Ltd. (Beijing, China). Dimethyl sulfoxide (DMSO, 99.8%) was provided by Sinopharm Chemical Reagent Co., Ltd. (Shanghai, China). All reagents were analytical grade reagents and were used without further purification.

### 2.2. Synthesis of P(VDF-HFP)-g-PGMA

A total of 0.5 g P(VDF-HFP) (VDF:HFP = 90:10) was dissolved in 10 mL DMSO solvent. Then, 3.65 mL GMA (27.5 mmol) and 10-phenylphenothiazine (0.0125 mmol) were added under N_2_ atmosphere. After stirring under N_2_ atmosphere for 30 min, the reaction mixture was placed under ultraviolet light (365 nm) at room temperature while cooling with a hair dryer. The product was precipitated in a mixed solution of methanol and water ((H_2_O/CH_3_OH) (V_1_:V_2_ = 1:1)) and washed three times with CH_3_OH. After drying, it was extracted with CHCl_3_ for 12 h. Finally, the obtained P(VDF-*co*-HFP)-*g*-PGMA was dried overnight under reduced pressure at 40 °C.

### 2.3. Preparation of P(VDF-co-HFP)-g-PGMA/CEC Composites

A certain amount of CEC was weighed and dissolved in DMSO solvent. The P(VDF-*co*-HFP)-*g*-PGMA was also dissolved in DMSO at room temperature. Then, the solution of CEC/DMSO and P(VDF-*co*-HFP)-*g*-PGMA/DMSO was mixed and stirred at room temperature for 10 h, and the mixed solutions were condensed and refluxed at a temperature of 100 °C for 24 h. The obtained solutions were poured onto a clean glass slide surface. The cast samples were dried in a blast drying furnace at 80 °C for 10 h and then transferred to a vacuum drying furnace at 80 °C for 12 h. Finally, the composites were dried in a 180 °C hydrothermal oven for 30 min and then removed and immersed in ice water for quenching.

### 2.4. Characterization

Fourier transform infrared spectroscopy (FTIR) was conducted using a PerkinElmer (Waltham, MA, USA) frontier infrared spectrometer in the spectral range of 4000–500 cm^−1^. Proton nuclear magnetic resonances (^1^H-NMR) and fluoride nuclear magnetic resonances (^19^F-NMR) were obtained using a Bruker (Billerica, MA, USA) (Ascend) 400 MHz instrument in DMSO-*d*_6_ containing tetramethylsilane (TMS) as the internal standard. Scanning electron microscopy (SEM) (JEOL, Tokyo, Japan) was performed with an ULTRA 55 field emission scanning electron microscope (FESEM) in the secondary electron mode with an accelerating voltage of 3 kV. The dielectric and alternating current (AC) conductive properties of the nanocomposites were measured on an HP4284A LCR meter (Hewlett-Packard, Palo Alto, CA, USA) in the frequency range of 100 Hz~1 MHz with 1 V at room temperature. Electric displacement–electric field (*D-E*) loops were obtained at 10 Hz using a Precision Premier II ferroelectric polarization tester (Radiant, Inc., Renton, WA, USA). Twelve specimens were used for the breakdown strength test for each sample. The energy storage performances were calculated according to the *D-E* results.

## 3. Result and Discussion

**Scheme 1:**
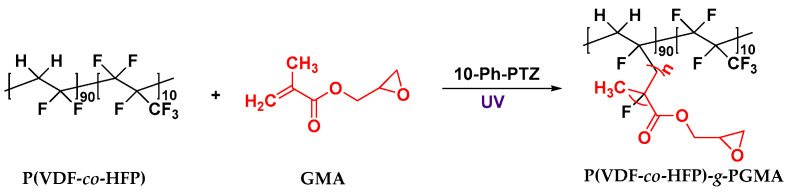
Synthesis of P(VDF-HFP)-*g*-PGMA with O-ATRP technology.

In this work, a “graft from” modification method via improved ATRP was proposed to prepare the metal-free P(VDF-HFP) graft copolymers (Scheme 1). *N*-phenylphenothiazine (PTH) were selected as the organocatalyst because of its features of easy obtainment and high catalytic activity under UV irradiation. Glycidyl methacrylate (GMA) was chosen as the functional monomer because of its high polar structure. Meanwhile, poly(glycidyl methacrylate) is miscible with P(VDF-HFP), which can reduce the crystallinity and crystal size of the fluoropolymer.

**Figure 1:**
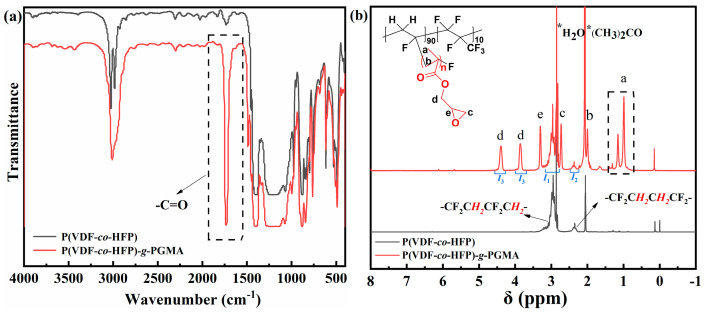
(**a**) FTIR and (**b**) ^1^H NMR of the P(VDF-*co*-HFP)-*g*-PGMA graft copolymers.

The structures of the original P(VDF-*co*-HFP) and P(VDF-*co*-HFP)-*g*-PGMA graft copolymers were characterized with Fourier transform infrared spectroscopy (FTIR), as shown in Figure 1a. The characteristic peak at 1726 cm^−1^ can be attributed to the carbonyl group of ester, which indicates the presence of the PGMA segment [6,25]. The epoxy bands around 905 cm^−1^ and 840 cm^−1^ overlap with P(VDF-*co*-HFP). Therefore, it can be preliminarily determined that the PGMA segment was successfully introduced into the main chain of P(VDF-*co*-HFP) [13].

In the ^1^H-NMR, as shown in Figure 1b, it can be seen that the multiple peaks appearing at 2.1–2.4 ppm and 2.7–3.2 ppm can be attributed to the head-to-head structure (*I*_2_, -CF_2_-C*H*_2_-C*H*_2_-CF_2_-) and head-to-tail structure (*I*_1_, -C*H*_2_-C*H*_2_-CF_2_-C*H*_2_-) of the VDF unit in the main chain of P(VDF-*co*-HFP), respectively. The labeled letters of the red line corresponded to the proton peaks of the PGMA structure in the ^1^H-NMR. For example, the multiple peaks at 0.8–0.9, 1.0–1.1 and 1.2–1.3 ppm can be assigned to the methyl proton. The multiple peaks at 3.7–4.1 and 4.3–4.7 ppm can be attributed to the methylene proton (*I*_3_, -COOCH_2_-) in PGMA [13,27].

According to the area of the characteristic peak in the ^1^H NMR in Figure 1b, the graft contents of the side chain of the PGMA segment in the polymerization product can be calculated as 16 mol% according to Equation (1) [13].

(1) $$\mathrm{GMA}\;\mathrm{grafted}\;(\mathrm{mol}\%)=\frac{I_{3}}{I_{1}+I_{2}}\times 90\;mol\%$$

**Figure 2:**
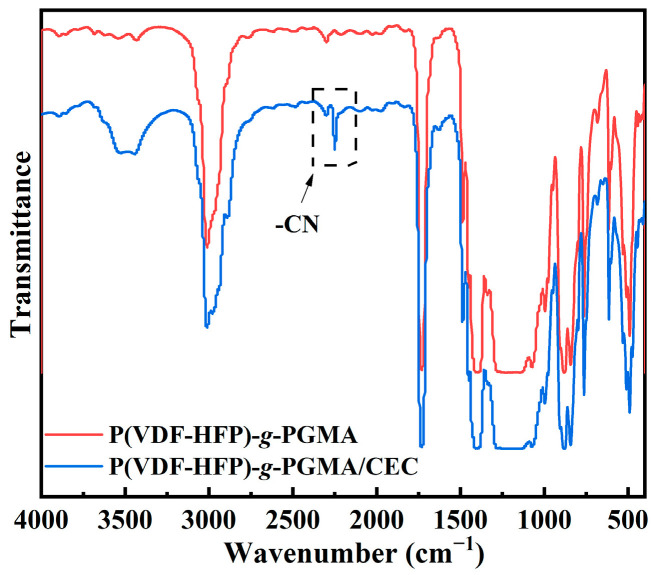
FTIR spectra of the P(VDF-*co*-HFP)-*g*-PGMA/CEC composite.

Then, due to the high dielectric constant and good compatibility of CEC, the P(VDF-*co*-HFP)-*g*-PGMA/CEC composite films were prepared using the solution cast method. A series of composite films compounded with 1 wt%, 3 wt% and 5 wt% CEC were successfully prepared. The structure of the P(VDF-*co*-HFP)-*g*-PGMA/CEC composite was characterized by FTIR, as shown in Figure 2. The characteristic peaks observed in the 850–1550 cm^−1^ range are typical for various carbon, polymer and polymer-like structures. Most of them are significantly wider than the PVDF-related lines observed in the 400–800 cm^−1^ range, which are ascribed to the disordered component of the material. The results presented show that the lines positioned at 870 and 970 cm^−1^ correspond to C-H bending. The 1060 and 1210 cm^−1^ lines are related to C-C, C-O and C-O-C bonds. The 1390 cm^−1^ position is typical both for bending vibrations of C-H bonds and for the stretching vibrations of C-C [28]. The characteristic peak at 2240 cm^−1^ can be attributed to the cyano group (-CN) of CEC, and the characteristic peak at 3500–3700 cm^−1^ can be attributed to the hydroxyl group (-OH) of CEC, which demonstrates the presence of CEC. In addition, the incorporation of CEC would not cause any structural modification in the original polymer matrix.

**Figure 3:**
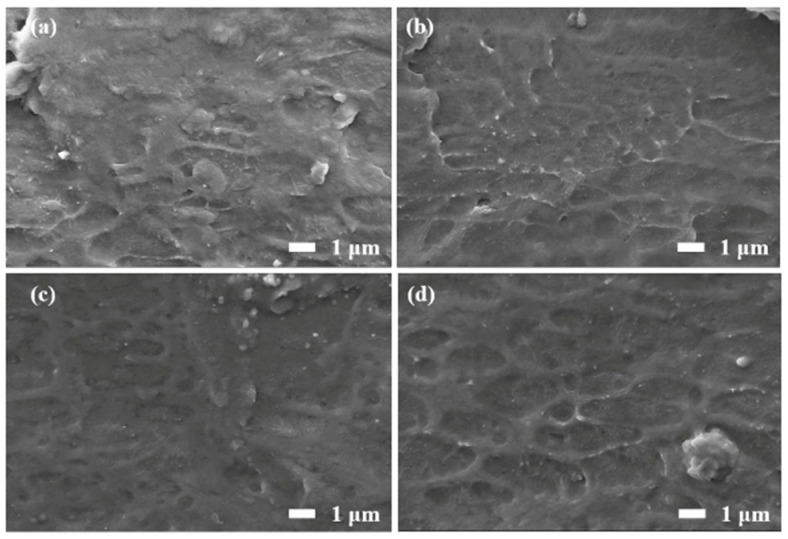
SEM images of the P(VDF-*co*-HFP)-*g*-PGMA/CEC composites: (**a**) 0 wt%; (**b**) 1 wt%; (**c**) 3 wt%; (**d**) 5 wt%.

Freeze-fractured cross-sectional SEM images that directly display the structure of the P(VDF-*co*-HFP)-*g*-PGMA/CEC composites are shown in Figure 3. The cross-sections of the samples were brittle fractured in liquid nitrogen and then dried. Moreover, the order of the SEM images are a compounding content of 0, 1, 3, and 5 wt% of the P(VDF-*co*-HFP)-*g*-PGMA/CEC composites. Among them, the P(VDF-*co*-HFP)-*g*-PGMA/1 wt% CEC composite in Figure 3b had better compatibility and dispersibility, whose interface looked more uniform. When the compounding contents of CEC were 3 wt% and 5 wt%, the polymer composites exhibited significant phase separation, especially as shown in Figure 3d.

**Figure 4:**
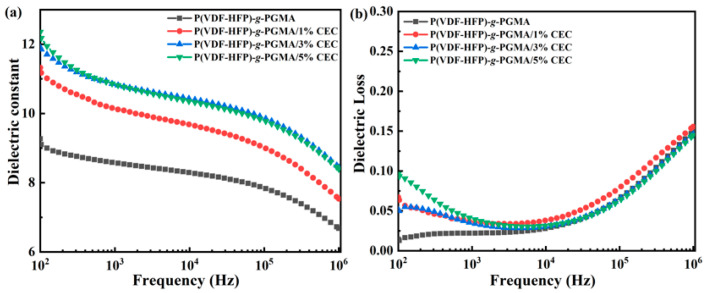
Frequency dependence of the (**a**) dielectric constant and the (**b**) dielectric loss of the P(VDF-*co*-HFP)-*g*-PGMA/CEC composites.

Figure 4 shows the dielectric properties of the P(VDF-*co*-HFP)-*g*-PGMA/CEC composites. Because of the capture of free charges, interface polarization can significantly increase the dielectric constant. Interface polarization mainly occurs in the low-frequency region and decreases with an increasing frequency [29,30]. At high frequencies, the dipole and space charge does not have enough time to rotate or move, resulting in a low dielectric constant of polymer dielectric films. It can clearly be seen from Figure 4a that due to the high dielectric constant of CEC, the dielectric constant of the polymer composites increased after the introduction of CEC. Moreover, as the compounding content of the CEC increased, the dielectric constant of the polymer composites also increased. As is shown in Figure 4b, after the introduction of CEC, the dielectric loss of the polymer composites at low frequencies also increased, which may be because of the increase in the interface polarization.

**Figure 5:**
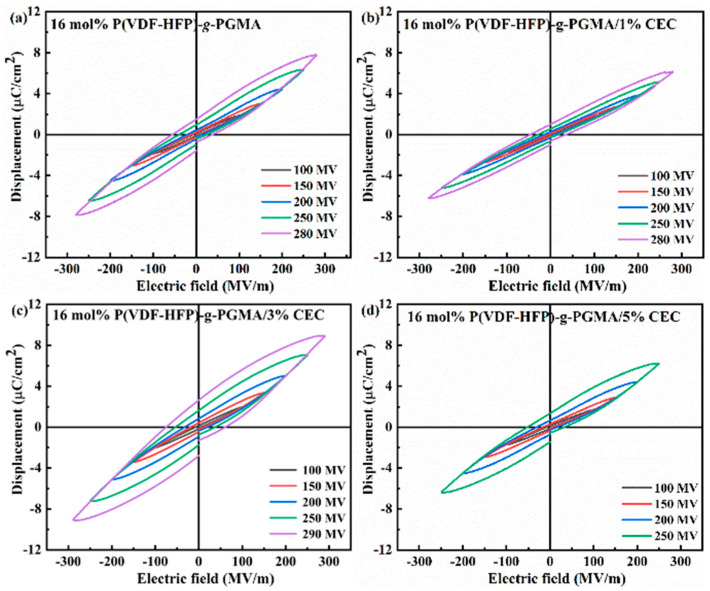
Bidirectional *D*-*E* loops of the P(VDF-*co*-HFP)-*g*-PGMA/CEC composites: (**a**) 0 wt%; (**b**) 1 wt%; (**c**) 3 wt%; (**d**) 5 wt%.

Figure 5 shows the bidirectional *D*-*E* loop of the P(VDF-*co*-HFP)-*g*-PGMA composites compounded with different mass fractions of CEC. The voltage started from an electric field of 100 MV/m and increased every 50 MV/m until the polymer composites broke down. Each curve in Figure 5 is a bidirectional *D*-*E* loop under its corresponding electric field. The bidirectional *D*-*E* loop became thinner after compounding at 1 wt% CEC, which indicates that 1 wt% CEC not only improved the dielectric constant of the polymer composites but also reduced the energy loss of the polymer composites. However, the bidirectional *D*-*E* loop gradually broadened after compounding with 3 wt% and 5 wt% CEC, which may be because the excessive amount of CEC resulted in a large loss of energy. In addition, it can also be seen that the polymer composite compounded with CEC had a higher maximum polarization (*P*_max_), which indicates that the introduction of CEC not only improved the dielectric constant of the polymer composites but also increased the maximum polarization. achieving the goal of improving the dielectric energy storage density.

**Figure 6:**
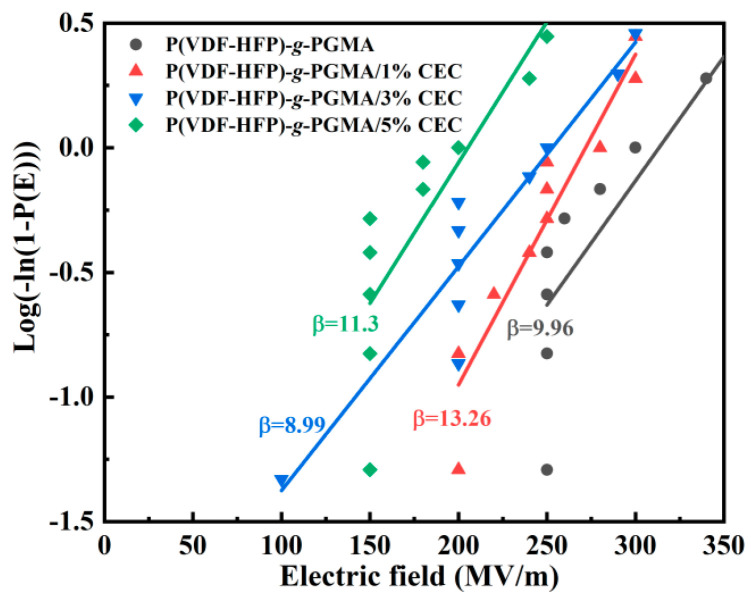
Weibull distribution of the breakdown strength of the P(VDF-*co*-HFP)-*g*-PGMA/CEC composites.

The breakdown strength was indirectly obtained through displacement hysteresis loop testing. As shown in Equation (2), the characteristic breakdown strength of the sample was calculated using the Weibull function [31,32,33].

(2) $$P\left(E\right)=1-{\exp\left(-\frac{E}{E_{b}}\right)}^{\beta }$$

where *P*(*E*) is the cumulative probability function of electric failure; *β* is a shape parameter related to data dispersion, and a high value of *β* represents a high level of reliability; *E* and *E*_b_ are the experimental breakdown strength and characteristic breakdown strength when the cumulative failure probability is 63.2% [34,35]. In this work, in order to obtain the Weibull distribution, each sample was tested 12 times for the maximum breakdown electric field. Figure 6 shows the Weibull distribution of the breakdown strength of the P(VDF-*co*-HFP)-*g*-PGMA/CEC dielectric composites. The breakdown strength of the P(VDF-*co*-HFP)-*g*-PGMA graft copolymer was between 250 MV and 350 MV. However, as the compounding amount of CEC increased, the breakdown properties of the P(VDF-*co*-HFP)-*g*-PGMA/CEC composites gradually decreased. The breakdown properties of the polymer composites compounded with the excessive CEC were poor, which may be because the excessive CEC contained a large number of hydroxyl groups, resulting in the poor breakdown properties of the polymer composites.

**Figure 7:**
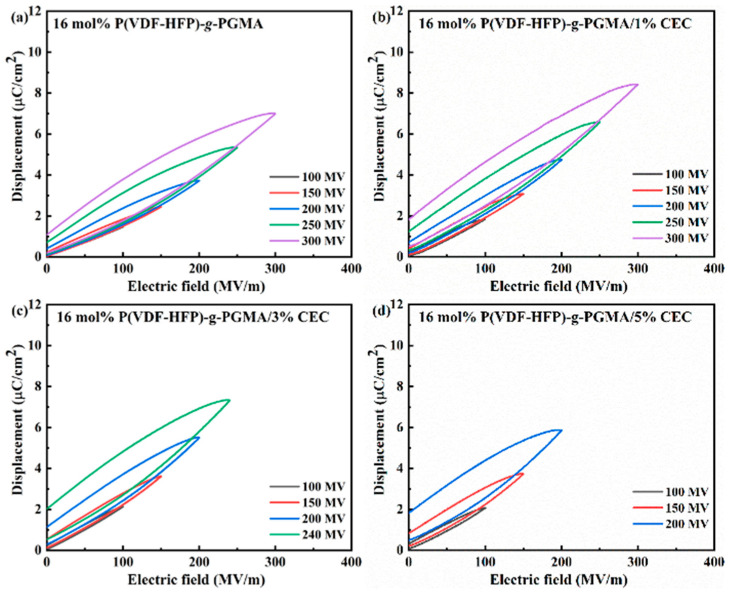
Unidirectional *D*-*E* loops of the P(VDF-*co*-HFP)-*g*-PGMA/CEC composites: (**a**) 0 wt%; (**b**) 1 wt%; (**c**) 3 wt%; (**d**) 5 wt%.

Figure 7 shows the unidirectional *D*-*E* loop of the P(VDF-*co*-HFP)-*g*-PGMA composites compounded with different mass fractions of CEC. Under the same electric field, the maximum polarization (*P*_max_) of the P(VDF-*co*-HFP)-*g*-PGMA/CEC polymer composites generally increased, which was attributed to the high dielectric constant of CEC. It is worth noting that the *P*_max_ of the compounded 1 wt% CEC polymer composites was the highest among the P(VDF-*co*-HFP)-*g*-PGMA/CEC polymer composites. For example, under an electric field of 300 MV/m, the *P*_max_ of the compounded 1 wt% CEC polymer composite was 8.42 μC/cm^2^, while for the pure graft copolymer it was 7.03 μC/cm^2^. This indicates that a combination of PVDF-based graft copolymers and high dielectric cellulose materials can maintain low remnant polarization while also improving the *P*_max_ of polymer composite materials. Therefore, the introduction of CEC into PVDF-based fluoropolymers can not only improve the compatibility of the two materials but also improve the *P*_max_ and energy storage capacity of polymer composites. This demonstrates the potential for achieving higher energy density capacitors.

Usually, the energy storage density of dielectric capacitors is determined with integration, as shown in Equation (3).

**Figure 8:**
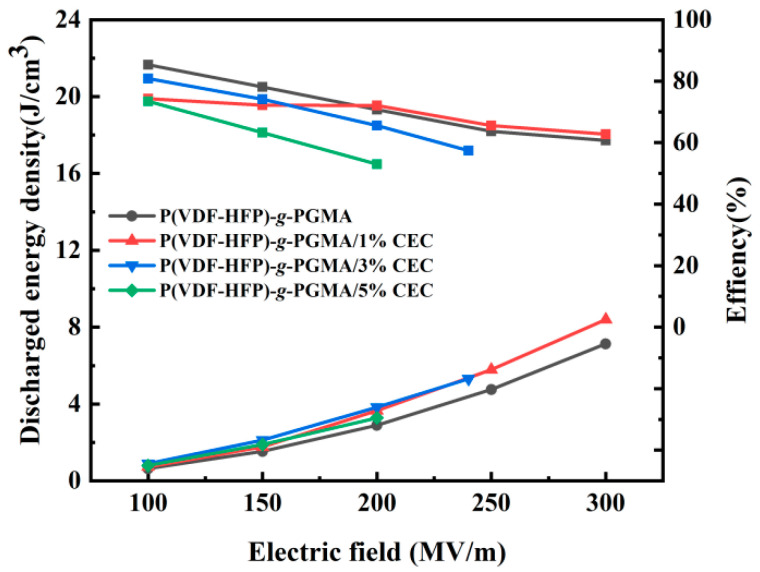
Comparative diagram of *U*_e_ and *η* of the P(VDF-*co*-HFP)-*g*-PGMA/CEC composites.

(3) U=∫E dD

where *E* is the applied electric field, *D* is the potential displacement, and the energy storage density can be calculated by the area enclosed by the *D*-*E* loop [2,16,36]. The energy storage density of the polymer composites is shown in Figure 8. The discharged energy storage density (*U*_e_) of the polymer composite compounded with 1 wt% CEC improved substantially compared with the pure graft copolymer P(VDF-*co*-HFP)-*g*-PGMA, and its charge and discharge efficiency (*η*) was not inferior to the P(VDF-*co*-HFP)-*g*-PGMA graft copolymer under an electric field above 200 MV/m. *η* is defined in Equation (4):

(4) $$\eta =\frac{U_{e}}{U_{e}+U_{i}}$$

where *U*_e_ is the discharge energy density, and *U*_i_ is the energy loss of the polymer composites [2]. The results show that the *U*_e_ of the P(VDF-*co*-HFP)-*g*-PGMA/1 wt% CEC polymer composite increased to 8.4 J/cm^3^ when the *U*_e_ of the pure graft copolymer was 7.12 J/cm^3^ under an electric field of 300 MV/m. In addition, the *η* of the graft copolymer was 60% under an electric field of 300 MV/m, while the *η* of the P(VDF-*co*-HFP)-*g*-PGMA/1 wt% CEC polymer composite was increased to 63%. The *U*_e_ of the polymer composites compounded with 3 wt% and 5 wt% CEC also improved, but their *η* decreased. Because excessive CEC did not form a good interaction with the PVDF-based fluoropolymers, it increased the loss of the polymer composites and reduced the charge and discharge efficiencies. It can be concluded that 1 wt% CEC can form a good interaction with PVDF-based fluoropolymers, thereby improving the dielectric properties and energy storage capacity of the polymer composites.

**Figure 9:**
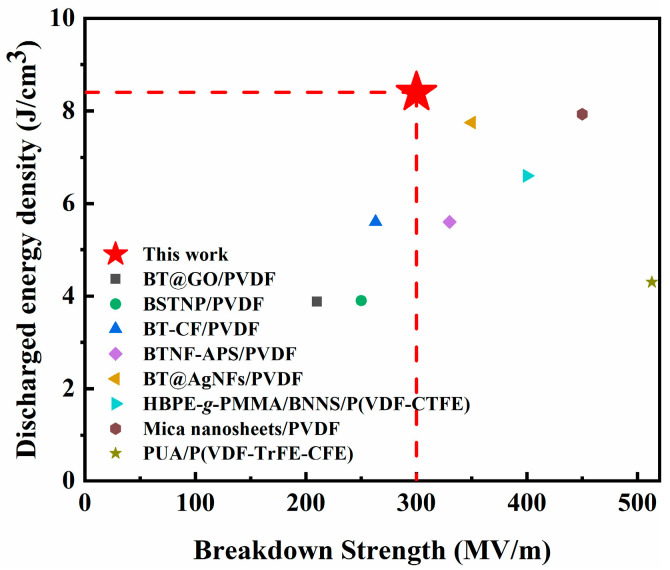
Comparison of the *U*_e_ of P(VDF-*co*-HFP)-*g*-PGMA/1 wt% CEC composites with other representative dielectric polymers [37,38,39,40,41,42,43,44].

Figure 9 presents a comparison of the *U*_e_ of the P(VDF-*co*-HFP)-*g*-PGMA/1 wt% CEC with other representative works of PVDF-based nanocomposites dielectrics. The superiority of the energy storage density of the P(VDF-*co*-HFP)-*g*-PGMA/1 wt% CEC is demonstrated by this comparison [37,38,39,40,41,42,43,44]. The full names of “BT@GO”, “BSTNP”, “BT-CF”, “BTNF-APS” and “BT@AgNFs” are “BaTiO_3_ nanoparticles@graphite”, “Ba_0.6_Sr_0.4_TiO_3_ nanoparticles”, “BaTiO_3_-CoFe_2_O_4_”, “BaTiO_3_ nanofibers-3-aminopropyltriethoxysilane” and “BaTiO_3_@Ag nanofibers”.

## 4. Conclusions

In this work, we developed P(VDF-HFP) graft copolymer/CEC composites to obtained all-organic composites dielectrics. The compatibility and dielectric constant of the composites were achieved simultaneously. Improved energy density was also obtained with this structure, which may be ascribed to the hydroxyl groups in CEC increasing the interaction with the polymer matrix. At the optimal condition, the P(VDF-*co*-HFP)-*g*-PGMA/1 wt% CEC polymer composite exhibited a high discharge energy density of 8.40 J/cm^3^ at 300 MV/m. This work contributes to the application of bio-based polymers serving as fillers in composites to yield high-performance dielectrics.

## Data Availability

Data will be made available upon request.
